# Self-reported sitting time is not associated with incidence of cardiovascular disease in a population-based cohort of mid-aged women

**DOI:** 10.1186/1479-5868-10-55

**Published:** 2013-05-07

**Authors:** Gerrie-Cor M Herber-Gast, Caroline A Jackson, Gita D Mishra, Wendy J Brown

**Affiliations:** 1 Centre for Longitudinal and Life course Research, School of Population Health, University of Queensland, Public Health Building, Herston Road, Herston, QLD 4006, Australia; 2 School of Human Movement Studies, University of Queensland, Brisbane, Australia

**Keywords:** Sitting time, Sedentary behaviour, Cardiovascular disease, Women

## Abstract

**Background:**

In Westernised societies adults are increasingly spending many hours each day in sedentary, low energy expenditure activities such as sitting. Although there is growing evidence on the relationship between television/screen time and increased cardiovascular disease mortality, very little is known about the association between total sitting time (in different domains) and cardiovascular disease incidence. We investigated this in a population-based cohort of mid-aged women in Australia.

**Findings:**

Data were from 6154 participants in the 1946–51 birth cohort of the Australian Longitudinal Study on Women’s Health who were free of cardiovascular disease at baseline. Survival analysis was used to determine the association between self-reported sitting time and cardiovascular disease incidence, determined through hospital diagnoses and cause of death data. During a mean (± SD) follow-up time of 9.9 ± 1.2 years, 177 cases of cardiovascular disease occurred. Mean sitting time (± SD) was 5.4 ± 2.6 hours a day. Sitting time was not associated with incident cardiovascular disease (adjusted hazard ratio 0.97, 95% CI 0.92 to 1.03). We found no interaction between physical activity and sitting time and cardiovascular disease.

**Conclusions:**

In mid-aged women sitting time does not appear to be associated with cardiovascular disease incidence. These findings are contrary to expectations, given the growing evidence of a relationship between sitting time and cardiovascular disease mortality. Research in this area is scarce and additional studies are needed to confirm or refute these findings.

## Findings

### Introduction

In Westernised societies many adults spend much of their day engaged in sedentary activities that involve low energy expenditure [1]. Recent reviews have found that sedentary behaviour is associated with increased risk of adverse health outcomes in adults, and that this is independent of physical activity level [2,3]. Several studies have demonstrated a relationship between sedentary behaviour and cause-specific mortality, including cardiovascular disease (CVD) mortality. A meta-analysis of eight studies found a 90% increased risk of CVD mortality when high versus low sedentary groups were compared [3]. However, the relationship with disease-specific incidence, including CVD incidence, is less clear and has been under-researched [2,3]. Of just four studies that have assessed sedentary behaviour and risk of *incident* CVD, [4-7] three found that higher television or screen-based viewing time was associated with significantly increased risk of CVD incidence. Only one of these studies defined sedentary behaviour as total sitting time [5]; it found an increased risk of CVD among women who spent at least 16 hours per day sitting, compared with those who sat for less than four hours per day [5]. Our aim was therefore to investigate the association between sitting time and CVD incidence in a prospective study of mid-aged women in Australia.

### Methods

We used data from participants in the Australian Longitudinal Study on Women’s Health (ALSWH), a national population-based study of women born in 1921–26, 1946–51 and 1973–78. Women were randomly selected from the Medicare database, which covers all citizens and permanent residents of Australia, including refugees and immigrants, with intentional oversampling of women living in rural and remote areas. Further details of the study are described elsewhere [8]. ALSWH was approved by the Human Research Ethics Committee of University of Newcastle, the Medical Research Ethics Committee of the University of Queensland and the Departmental Ethics Committee of the Australian Government Department of Health and Ageing. The study complies with the Helsinki Declaration.

Our focus was on the 1946–51 cohort, who were surveyed in 1996, 1998 and every three years thereafter. In these analyses, we included those who responded to survey three in 2001 (n = 10,628), and who lived in the states of New South Wales (NSW), Queensland (QLD) or Western Australia (WA), giving a total of 6739 women. Linked hospital admission data were not available for the other states, and were not available for NSW or QLD prior to 2000. We excluded women with prevalent heart disease or stroke, based on 2001 self-report and hospital admission data (n = 327), and with missing data on sitting time (n = 173) or any of the covariates (n = 85), thereby including data from 6154 women in the analyses.

Sitting time was assessed by asking: ‘How many hours each day do you typically spend sitting down while doing things like visiting friends, driving, reading, watching television or working at a desk or computer (a) on a usual week-day and (b) on a usual weekend-day?’ Sitting time data were cleaned using protocols developed by van Uffelen et al. and mean sitting time in hours/day was calculated as ((weekday sitting × 5 + weekend day sitting × 2)/7) [9].

Covariates included demographic (age, education level and marital status) and lifestyle factors (body mass index (BMI), smoking status, physical activity [10] and alcohol consumption, defined in light of the Australian National Health and Medical Research Council guidelines [11]), categorised as shown in Table 1.

**Table 1:** Demographic and health characteristics of the participants, by sitting time and CVD

		**Sitting time**				
	**Total group**	**Low**	**Moderate**	**High**	**No CVD**	**CVD**
	**(n = 6,154)**	**(n = 1,860)**	**(n = 2,232)**	**(n = 2,062)**	**(n = 5,977)**	**(n = 177)**
**Age**	52.5 ± 1.5	52.5 ± 1.5	52.5 ± 1.5	52.5 ± 1.4	52.5 ± 1.5	52.8 ± 1.4
**Sitting time, hours/day**	5.4 ± 2.6	2.7 ± 0.8	4.9 ± 0.7	8.4 ± 1.8	5.4 ± 2.6	5.2 ± 2.3
**Follow-up time, months**	118.6 ± 14.5	118.8 ± 13.5	118.2 ± 16.0	119.1 ± 13.7	120.2 ± 10.0	67.0 ± 34.5
**CVD case, yes**	177 (2.9)	48 (3.1)	57 (3.0)	47 (2.7)		
**Educational level**						
No formal qualifications	914 (14.9)	307 (16.5)	351 (15.7)	256 (12.4)	888 (14.9)	26 (14.7)
School or leaving certificate	3,130 (50.9)	964 (51.8)	1,113 (49.9)	1,053 (51.1)	3,045 (51.0)	85 (48.0)
Trade/apprenticeship or higher education	2,110 (34.3)	589 (31.7)	768 (34.4)	753 (36.5)	2,044 (34.2)	66 (37.3)
**BMI (kg/m**^**2**^**)**						
Underweight (<18.5)	96 (1.6)	36 (1.9)	24 (1.1)	36 (1.8)	93 (1.6)	3 (1.7)
Healthy weight (18.5-24.9)	2,678 (43.5)	912 (49.0)	967 (43.3)	799 (38.8)	2,606 (43.6)	72 (40.7)
Overweight (25–30)	2,014 (32.7)	588 (31.6)	760 (34.1)	666 (32.3)	1,953 (32.7)	61 (34.5)
Obese (>30)	1,366 (22.2)	324 (17.4)	481 (21.6)	561 (27.2)	1,325 (22.2)	41 (23.2)
Physical activity*						
Nil/sedentary	1,097 (17.8)	300 (16.1)	362 (16.2)	435 (21.1)	1,057 (17.7)	40 (22.6)
Low	2,297 (37.3)	690 (37.1)	793 (35.5)	814 (39.5)	2,231 (37.3)	66 (37.3)
Moderate	1,232 (20.0)	369 (19.8)	462 (20.7)	401 (19.5)	1,206 (20.2)	26 (14.7)
High	1,528 (24.8)	501 (26.9)	615 (27.6)	412 (20.0)	1,483 (24.8)	45 (25.4)
**Smoking status**						
Non-smoker	3,727 (60.6)	1,153 (62.0)	1,361 (61.0)	1,213 (58.8)	3,627 (60.7)	100 (56.5)
Ex-smoker	1,546 (25.1)	458 (24.6)	549 (24.6)	539 (26.1)	1,500 (25.1)	46 (26.0)
Current smoker	881 (14.3)	249 (13.4)	322 (14.4)	310 (15.0)	850 (14.2)	31 (17.5)
**Alcohol consumption†**						
Low risk drinker	3,285 (53.4)	943 (50.7)	1,184 (53.1)	1,158 (56.2)	3,190 (53.4)	95 (53.7)
Non-drinker	782 (12.7)	256 (13.8)	295 (13.2)	231 (11.2)	762 (12.8)	20 (11.3)
Rarely drinker	1,717 (27.9)	549 (29.5)	631 (28.3)	537 (26.0)	1,660 (27.8)	57 (32.2)
Risky drinker	370 (6.0)	112 (6.0)	122 (5.5)	136 (6.6)	365 (6.1)	5 (2.8)

*Defined in MET. Min/week as: Nil/sedentary – <40 MET; low – 40–299; moderate – 300–599; high - ≥600.

†‘Risky drinkers’ (15 to 28 drinks per week) and ‘High risk drinkers’ (More than 28 drinks per week) were grouped together. For women identified as low risk (<15 drinks per week) by the NHMRC guidelines, we separately categorised those classified as low-risk drinkers from those reporting that they drink only rarely.

Data on morbidity were obtained from hospital admitted patient discharge data from 2001 to 2010 for NSW, 2001–2011 for WA and QLD public hospitals, and from July 2007 through 2011 for QLD private hospitals. Non-fatal and fatal CVD events were identified using International Statistical Classification of Diseases and Related Health Problems codes (9th revision (ICD-9): 410–414, 430–438; or 10th revision (ICD-10): I20-I25, I60-I67, and I69). If multiple CVD events occurred, the first diagnosis was taken as the endpoint. Women were followed from the month of return of survey three (2001) until the first nonfatal CVD event, death, or were censored at 31 December 2010 (NSW), 16 June 2011 (WA) or 16 December 2011 (QLD). Information on vital status was available through linkage to the National Death Index.

Statistical analyses were performed using SAS 9.2 software. Baseline characteristics were described according to sitting time tertiles by means and SDs for normally distributed continuous variables and numbers and percentages for categorical variables. Cox proportional hazards models were used to estimate hazard ratios (HRs) and 95% confidence intervals (CIs) of CVD risk for continuous data and tertiles of sitting time using the lowest tertile as the reference.

For univariable analyses, a model was fitted for sitting time and risk of CVD (model 1). Multivariable models included age, education, smoking and alcohol consumption (model 2), physical activity (model 3) and BMI (model 4). We also formally tested effect modification by adding interaction terms between sitting time and physical activity. Differences with P-values <0.05 were considered to be statistically significant.

### Results

Mean age at baseline (2001) was 52.5 ± 1.5 years. Mean sitting time was 5.4 ± 2.6 hours a day. Compared with women in the lowest category of sitting time, those with the highest sitting time were more educated and more likely to be obese, low risk alcohol drinkers, and less physically active (Table 1). During a mean follow-up of 9.9 ± 1.2 years, 177 CVD events occurred, of which four were fatal.

Sitting time (as a continuous variable) was not associated with risk of CVD; this pattern did not change after adjustment for potential confounders (HR 0.97, 95% CI 0.92, 1.03); Table 2, model 4). In anticipation that only high levels of sitting time may be associated with CVD risk, we also analysed sitting time as a categorical variable, but found no associations with CVD (Table 2).

**Table 2:** Hazard ratios (95% CIs) for sitting time and cardiovascular disease risk in the total study population (N = 6,154)

	**Sitting time continuous**	**Sitting time in categories**		
		**Low**	**Moderate**	**High**
No. of cases	**177**	**48**	**57**	**47**
Model 1	0.97 (0.92,1.03)	Ref	1.01 (0.71,1.45)	0.91 (0.63,1.32)
Model 2	0.98 (0.92,1.03)	Ref	1.03 (0.72,1.48)	0.93 (0.64,1.35)
Model 3	0.97 (0.92,1.03)	Ref	1.03 (0.72,1.48)	0.91 (0.62,1.32)
Model 4	0.97 (0.92,1.03)	Ref	1.03 (0.72,1.47)	0.90 (0.62,1.32)
Interaction with PA	0.76	0.84		

Model 1: crude; Model 2: adjusted for age, education, smoking, alcohol consumption; Model 3: Model 2 and physical activity; Model 4: Model 3 and BMI.

Formal tests for interaction with physical activity revealed no significant interactions (p-values for effect modification were 0.76 and 0.84 for the continuous and categorical sitting time variables respectively).

### Discussion

We found no association between sitting time and incidence of CVD among mid-aged women.

These findings are contrary to those expected, given that previous studies have shown a positive association between sedentary behaviour and CVD incidence [4-7], and between total sitting time and CVD mortality [12-14]. Differences in the exposure measure may partly explain this difference. All but one of the four studies of CVD incidence measured television viewing time [4,7], or ‘screen time’ [6]. This type of sedentary behaviour may be associated with a different level of CVD risk compared with total sitting time, since it may be associated with long periods of uninterrupted sitting, or unhealthy eating patterns. Only one study (which principally compared relationships between walking and vigorous activity with CVD in women aged 50–79 at baseline) asked participants about *total* sitting time [5], as we did. This study found that sitting for 16 hours or more per day increased CVD risk by 68% compared with sitting for less than four hours per day. Other durations of sitting were not associated with increased CVD risk.

Most previous studies similarly used hospital records and/or routinely collected mortality data to define CVD. However, the definition of CVD was generally much broader than in our study, which may partly explain the inconsistency in findings. The distribution of total sitting time in our study also differs, in that our participants appear to be less sedentary than in some other study populations [5,12]. The majority of women were in the middle range of sitting time, and even the highest tertile group had a mean sitting time of only 8.4 hours/day. Therefore, it is possible that overall sitting time was perhaps too low in our cohort to detect an impact on CVD incidence within the follow-up period. Differences in age of study population and physical activity levels do not appear to explain contrasting findings with previous studies on CVD incidence. Most participants were similarly middle-aged, and the physical activity levels, albeit measured in different ways, appear to be comparable. Among studies of sitting time and CVD mortality, participants included elderly people in two studies [12,13], and although a third study included a comparable age-group, the association between sitting time and mortality was not adjusted for confounders [14]. Interestingly, recent results from our group have shown that prolonged sitting time is associated with all-cause mortality in the cohort born 1921–1926 [15] and with breathing difficulties and chest pain [16] (which may be symptomatic of CVD) in the 1946–1951 cohort of ALSWH.

Our study has a number of strengths. The study population was large and community-based which improves the generalizability of our findings to mid-aged women. We ascertained CVD outcomes using an objective measure based on hospital admission and cause of death data. Studies using similar registries have shown that 89% of suspected CVD outcomes are confirmed against internationally agreed criteria [17], so that outcome misclassification is probably not a major issue in our study.

There are a few limitations. First, sitting time was based on self-report and bias may have been introduced if measurement error was related to occurrence of CVD. However, it is likely that any measurement error was random, which may have led to underestimation of any association between sitting time and CVD. Similar sitting time questions are used in the International Physical Activity Questionnaire, which, in women, have been shown to have good reliability and moderate criterion validity against accelerometers (<100 counts/min) [18]. Our study population was on average aged 52.5 years at baseline and despite following women for an average of 9.9 years, it may take longer for sitting time to impact on CVD in this particular age group. This may explain why sitting time was associated with possible CVD symptoms [16], but not CVD events. Second, the number of CVD events was relatively low. However, with this study size we were powered to detect a minimum relative risk of 1.17, which is much smaller than that observed in studies of sitting time and CVD mortality. Third, our study population included women only and we cannot assume that similar results would be observed in men. Finally, we did not have country-wide hospital admission data and thus would not have identified a small number of CVD outcomes that may have occurred outside included states.

In this study we did not find an association between sitting time and CVD incidence. Further large prospective population-based studies in different settings, age-groups and study populations, with objective measurement of both sitting time and CVD incidence, sufficiently long follow-up and a distinction between leisure time and occupational sitting time, are needed to confirm or refute our findings.

## Abbreviations

CVD: Cardiovascular disease; HR: Hazard ratio; CI: Confidence interval.

## Competing interests

The authors declare that they have no competing interests.

## Authors’ contributions

GCMHG, CJ, WB and GM conceived and designed the study. GCMHG and CJ performed the analyses, interpreted the results and co-drafted the manuscript. WB and GM interpreted the results and commented on the draft manuscript. All authors read and approved the final manuscript.
